# The Impact of Small Time Delays on the Onset of Oscillations and Synchrony in Brain Networks

**DOI:** 10.3389/fnsys.2021.688517

**Published:** 2021-07-05

**Authors:** Isam Al-Darabsah, Liang Chen, Wilten Nicola, Sue Ann Campbell

**Affiliations:** ^1^Department of Mathematics, University of Manitoba, Winnipeg, MB, Canada; ^2^Department of Applied Mathematics, Centre for Theoretical Neuroscience, University of Waterloo, Waterloo, ON, Canada; ^3^Hotchkiss Brain Institute, Cumming School of Medicine, University of Calgary, Calgary, AB, Canada

**Keywords:** synchronization, time delay, Wilson-Cowan network, homeostatic synaptic plasticity, master stability function, network neuroscience, connectomes

## Abstract

The human brain constitutes one of the most advanced networks produced by nature, consisting of billions of neurons communicating with each other. However, this communication is not in real-time, with different communication or time-delays occurring between neurons in different brain areas. Here, we investigate the impacts of these delays by modeling large interacting neural circuits as neural-field systems which model the bulk activity of populations of neurons. By using a Master Stability Function analysis combined with numerical simulations, we find that delays (1) may actually stabilize brain dynamics by temporarily preventing the onset to oscillatory and pathologically synchronized dynamics and (2) may enhance or diminish synchronization depending on the underlying eigenvalue spectrum of the connectivity matrix. Real eigenvalues with large magnitudes result in increased synchronizability while complex eigenvalues with large magnitudes and positive real parts yield a decrease in synchronizability in the delay vs. instantaneously coupled case. This result applies to networks with fixed, constant delays, and was robust to networks with heterogeneous delays. In the case of real brain networks, where the eigenvalues are predominantly real, owing to the nearly symmetric nature of these weight matrices, biologically plausible, small delays, are likely to increase synchronization, rather than decreasing it.

## 1. Introduction

Biological systems often form intricate and highly interconnected networks. Examples include the chemical reaction networks present within a single cell at the small scale (Kitano, 2002), the spread of disease through social networks (Keeling and Eames, 2005) or ecological networks across entire biomes or even the planet itself at the large scale (Montoya et al., 2006). Yet, one of the critical defining features in these networks is that communication from putative nodes is seldom instantaneous, and is often plagued by delays. Nowhere is this clearer than in the human brain, an intricate network of neurons limited by the slow propagation speed of action potentials or spikes, which can take up to milliseconds to transmit information across areas (Roxin et al., 2005; Ghosh et al., 2008; Deco et al., 2009).

This seems unusual when we consider the readily synchronizable nature of brain matter. For example, pathologically strong synchrony exists in neurological disorders such as epilepsy despite the presence of time-delays (Uhlhaas and Singer, 2006). Beyond pathological states, weakly synchronized brain areas are normal and even necessary states for the functioning of brain networks during a variety of tasks (Varela et al., 2001). Indeed, the presence of delays alone can have variable impacts on synchronization with synchronizability determined by (1) the topology of the network, (2) the dynamics of the nodes, and (3) the nature of the delays.

We investigated how these three forces would interact with computational modeling in networks of homeostatically-coupled Wilson-Cowan (WC) nodes (Wilson and Cowan, 1972; Destexhe and Sejnowski, 2009; Vogels et al., 2011; Cowan et al., 2016; Hellyer et al., 2016; Nicola et al., 2018). In this model, each node can be interpreted as a population of excitatory and inhibitory neurons. The nodes are stabilized onto a steady-state equilibrium with a homeostatic, dynamically adjusted weight which strives to maintain a stable firing rate in each population (Nicola et al., 2018; Nicola and Campbell, 2021). However, the homeostatic adjustment of weights can also lead to more complex dynamics, such as mixed mode oscillations and chaos, which and lead to desynchronization of the nodes (Nicola et al., 2018; Nicola and Campbell, 2021). Here, we show how the presence of small time delays in the coupling influences dynamic behavior and synchronization in comparison to the instantaneously coupled networks. We limit our study to delay magnitudes that are biologically relevant; these are small in comparison with other time scales in the model. First, we find that the induction of oscillations (via a Hopf bifurcation) requires larger global coupling strengths in the delay coupled network vs. the instantaneously coupled system. Second, we find that for a sufficiently large delay, the system readily loses all non-relaxation oscillator solutions (period doubling cascades, mixed-mode dynamics, chaos) past the Hopf-bifurcation. Third, by applying a master-stability formalism to these networks, we find that synchronization is dependent on the underlying graph and the nature of the time-varying synchronized solutions. The delays decreased the synchronizability of graphs with large complex eigenvalues (with postive real parts) while increasing the synchronizability of graphs with purely real eigenvalues, as in the case of DTI-derived connectomes (Bullmore and Sporns, 2009). For small delays, synchronization could occur for chaotic or other complex solutions as in the nondelayed case. For sufficiently large delays, however, synchronization was always associated with oscillatory solutions. This general portrait of the interactions between network topology, dynamics, and delays was also robust to delay heterogeneity throughout the network. Thus, we find that rich dynamics and variable synchronizability with different graph structures.

## 2. Materials and Methods

### 2.1. Model Equations

**Figure 1:**
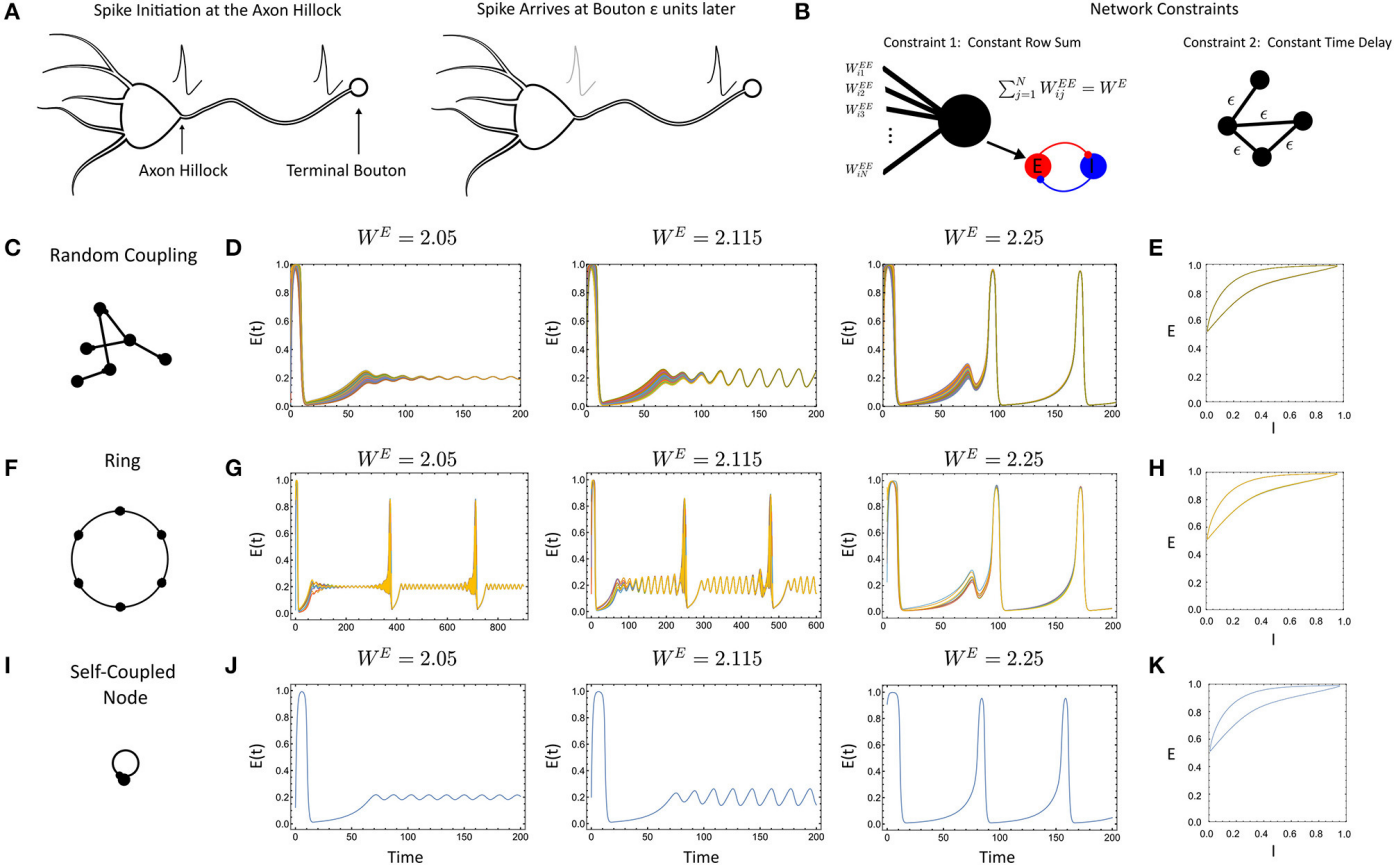
**(A)** Communication delays in neural networks are caused by the non-instantaneous transmission of an action potential down an axon. The spike is initiated at the axon hilock and arrives at the terminal bouton after a period of time. **(B)** The primary constraints in system (Equation 1), the row-sum of the weights normalizes to *W*^*E*^ and all the delays are constant. **(C)** Randomly-coupled networks with constant delay. **(D)** Simulation for increasing *W*^*E*^ **(E)** Phase portrait of [*E*(*t*), *I*(*t*)] for the network after synchronization for *W*^*E*^ = 2.25. **(F)** Ring networks. **(G)** Simulated ring network with *N* = 8 nodes for increasing *W*^*E*^ **(H)** Same as **(E)** except for ring topology. **(I)** The single, delay coupled node. **(J)** Simulations of the single, delay coupled node. **(K)** Same as **(E,H)** only for the single, delay coupled node. For all simulations, the parameter values were: ϵ = 0.1, *p* = 0.2, τ_1_ = 1, τ_2_ = 5, *W*^*IE*^ = 1, and *a* = 5.

To model the system we use a Wilson-Cowan network with homeostatic regulation of the inhibitory connection weight due to Vogels et al. (2011), Hellyer et al. (2016), Nicola et al. (2018), and Nicola and Campbell (2021). We introduce a time delay in the excitatory connections between the nodes (Figure 1A).

(1) $$\begin{array}{l} \begin{array}{l} \tau_{1}\frac{dE_{k}}{dt}=-E_{k}+\phi \left(\sum_{j=1}^{N}W_{kj}^{EE}E_{j}\left(t-\epsilon_{kj}\right)-W_{k}^{EI}I_{k}\right)\\ \frac{dI_{k}}{dt}=-I_{k}+\phi \left(W^{IE}E_{k}\right)\\ \tau_{2}\frac{dW_{k}^{EI}}{dt}=I_{k}\left(E_{k}-p\right) \end{array} \end{array}$$

*E*_*k*_ is the activity of the excitatory population of neurons within the *k*th node, *I*_*k*_ is the activity of the inhibitory population in the *k*th node, $W_{k}^{EI}$ is the homeostatically adjusted inhibitory weight of the *k*th node and *W*^*IE*^ is the fixed excitatory weight of the *k*th node. $W_{kj}^{EE}>0$ are the (fixed) excitatory weights and ϵ_*kj*_ is the time delay between nodes. The function ϕ is a sigmoidal transfer function which we take to be the logistic function:

(2) $$\begin{array}{l} \phi \left(x\right)=\frac{1}{1+\exp\left(-ax\right)} \end{array}$$

where *a* controls the steepness of the sigmoid, while the sigmoid itself determines the proportion of the population of neurons which is active in node *k*.

We use the parameter values as described in Nicola et al. (2018): *p* = 0.2, *a* = 5, τ_1_ = 1, τ_2_ = 5. The values of *W*^*IE*^ and $W_{kj}^{EE}$ are varied. To choose an appropriate value for the time delay, ϵ, note that in Equation (1) time has already been scaled by the timescale of the inhibitory population, τ_*I*_ (Nicola et al., 2018). This means that the delays are also scaled $\epsilon_{ij}=\frac{T_{ij}}{\tau_{I}}$. From Hellyer et al. (2016) we find values of *T*_*ij*_ in the range 1 − 14 ms and τ_*I*_ = 20 ms, which yields ϵ_*ij*_ in the range 0.05 − 0.7.

In our work, we consider two primary constraints on this system (Figure 1B). First, the row-sum of the weight matrix ***W***^*EE*^ is constant:

(3) $$\begin{array}{l} \overset{N}{\underset{j=1}{\sum }}W_{kj}^{EE}=W^{E},\;k=1,2,\ldots N \end{array}$$

where the parameter *W*^*E*^ acts as the global coupling strength of the entire system. The second constraint is that the delays are homogeneous throughout the network:

(4) $$\begin{array}{l} \epsilon_{kj}=\epsilon ,\;\forall k,j \end{array}$$

However, in Figure 4 we consider the impact of heterogeneous delays by choosing the delays ϵ_*kj*_ value from a Beta distribution with an average of ϵ.

### 2.2. The Synchronous Solution and the Single, Self-Coupled Node

The model (Equation 1) with the constraints (Equations 3, 4) admits a synchronous solution (*E*_*k*_, *I*_*k*_, $W_{k}^{EI}$) = (*E*_*s*_(*t*), *I*_*s*_(*t*), $W_{s}^{EI}$(*t*)), *k* = 1, …, *N*. The functions (*E*_*s*_(*t*), *I*_*s*_(*t*), $W_{s}^{EI}$(*t*)) satisfy the equations for a single, isolated node with delayed, self-coupling

(5) $$\begin{array}{l} \tau_{1}\frac{dE}{dt}=-E+\phi \left(W^{E}E\left(t-\epsilon \right)-W^{EI}I\right) \end{array}$$

(6) $$\begin{array}{l} \frac{dI}{dt}=-I+\phi \left(W^{IE}E\right) \end{array}$$

(7) $$\begin{array}{l} \tau_{2}\frac{dW^{EI}}{dt}=I\left(E-p\right) \end{array}$$

The self-coupling arises from the analysis of the synchronous solution and is independent of whether there is self-coupling in the full model. See Supplementary Materials Section 1 for details.

Thus, the synchronous solution of Equation 1 can be described by analyzing the behavior of model for a single, self-coupled node (Equations 5–7). For example, this model has an equilibrium solution which yields the following equilibrium solution of the full model

$$\left({\bar{E}}_{k},{\bar{I}}_{k},{\bar{W}}_{k}^{EI}\right)=\left(p,\phi \left(W^{IE}p\right),\frac{W^{E}p-\phi^{-1}\left(p\right)}{\phi \left(W^{IE}p\right)}\right),\;k=1,\ldots N.$$

Analysis of the linearization of Equation 1 about this equilibrium point shows that a Hopf bifurcation occurs for a sufficiently strong global coupling strength, *W*^*E*^, as a function of the excitatory-to-inhibitory coupling parameter *W*^*IE*^,

$$W_{Hopf}^{E}=g\left(W^{IE}\right)$$

This Hopf-bifurcation curve can be approximated via a perturbation analysis in the limit of small delays (ϵ ≪ 1, see Supplementary Materials Section 2).

### 2.3. Master Stability Function

The Master stability function was first developed to study synchronization in large networks of coupled oscillators without time delay (Pecora and Carroll, 1990). The derivation for systems with time delays has been described in Dhamala et al. (2004), Choe et al. (2010), and Flunkert et al. (2010). The application to the model (Equation 1) is almost identical to that described in Nicola and Campbell (2021) (see Supplementary Materials Section 3).

Assuming that *W*^*EE*^ is diagonalizable, the linear (local) stability of the synchronized solution $\left(E_{k},I_{k},W_{k}^{EI}\right)=\left(E_{s}\left(t\right),I_{s}\left(t\right),W_{s}^{EI}\left(t\right)\right),\;k=1,\ldots ,N$ of the model (Equation 1) can be determined by studying the three dimensional linear system

(8) $$\begin{array}{l} \begin{array}{l} \tau_{1}\frac{d\eta_{x}}{dt}=-\eta_{x}+M_{s1}\left(t\right)\left(\hat{r}\eta_{x}\left(t-\epsilon \right)-I_{s}\left(t\right)\eta_{z}-W_{s}^{EI}\left(t\right)\eta_{y}\right)\\ \;\frac{d\eta_{y}}{dt}=-\eta_{y}+M_{s2}\left(t\right)\eta_{x}\\ \tau_{2}\frac{d\eta_{z}}{dt}=\left(E_{s}\left(t\right)-p\right)\eta_{y}+I_{s}\left(t\right)\eta_{x} \end{array} \end{array}$$

where $M_{s1}\left(t\right)=\phi^{\prime }\left(W^{E}E_{s}\left(t-\epsilon \right)-W_{s}^{EI}\left(t\right)I_{s}\left(t\right)\right)$ and $M_{s2}\left(t\right)=W^{IE}\phi^{\prime }\left(W^{IE}E_{s}\left(t\right)\right)$, and $\hat{r}$ is an eigenvalue of *W*^*EE*^. The Master stability function, λ(*r*) is typically defined as follows. For a given *r* ∈ ℂ if the trivial solution of (Equation 8) asymptotically stable, then λ(*r*) < 0. If it is unstable then λ(*r*) > 0. A standard approach is to define λ(*r*) be the maximal Lyapunov exponent of the system (Equation 8). The MSF is then used to define a *region of stability* in the complex plane, corresponding to all values of *r* for which λ(*r*) < 0. If all eigenvalues of *W*^*EE*^ lie inside this region then the synchronous solution of Equation 1 is locally asymptotically stable. Finally, we remark that we primarily consider the scaled eigenvalues, $r_{k}=\frac{{\hat{r}}_{k}}{W^{E}}$ for all numerical simulations and plots, thereby allowing us to compare eigenvalues on the unit circle across global coupling strengths.

### 2.4. Numerical Methods

We use the commands ParametricNDSolveValue in *Wolfram Mathematica* and NDSolveValue to simulate the system (1) with homogeneous and heterogeneous delays. We used the numerical continuation package DDE-Biftool (Engelborghs et al., 2001) to compute Hopf bifurcation curves and period doubling curves for the model (Equations 5–7) in the *W*^*IE*^, *W*^*E*^ parameter space.

###  Numerically Implementing the Master Stability Function for a Delay Differential System

The Master Stability Function (MSF) approach for a generic delay differential system

(9) $$\begin{array}{l} \frac{dx}{dt}=F\left(x\left(t-\epsilon \right),x\left(t\right)\right) \end{array}$$

is performed by first discretizing the delay-differential system:

(10) $$\begin{array}{l} \frac{dx_{1}}{dt}=F\left(x_{m},x_{1}\right) \end{array}$$

(11) $$\begin{array}{l} \frac{dx_{n}}{dt}=\left(x_{n+1}\left(t\right)-x_{n-1}\left(t\right)\right)\cdot \frac{m}{2\epsilon },\;n=1,2,\ldots m-1 \end{array}$$

(12) $$\begin{array}{l} \frac{dx_{m}}{dt}=\left(x_{m-1}\left(t\right)-x_{m}\left(t\right)\right)\cdot \frac{m}{\epsilon }, \end{array}$$

as in Farmer (1982) and Lakshmanan and Senthilkumar (2011).

This approximation is applied to the linearized system with delays (Equation 8) which reduces the original system of 3*N* delay differential equations to a system of 3*Nm* ordinary differential equations. Then, the classical MSF approach via computing the Lyapunov exponents of the reduced variational equations is now immediately applicable as the resulting network consists of coupled ordinary differential equations. Details of the implementation can be found online (see Code Availability Statement). The value of *m* = 10 discretization points was taken.

To supplement this approach, we performed numerical simulations of the linear delay differential equation system (Equation 8) and tracked whether solutions decayed to zero or not. This was then used to define the MSF. This yielded results consistent with those from the discretized DDE.

## 3. Results

###  Delay Coupled Wilson-Cowan Systems Can Still Synchronize

With the initial network constructed, we first sought to determine what impacts the delay would have, if any, by comparison with results for the instantaneously coupled network. To assess this, we conducted an initial barrage of simulations with randomly-coupled networks (Figures 1C–E), ring networks (Figures 1F–H), and the single, self-coupled node with delay (Figures 1I–K). Simulations for larger delay (ϵ = 0.3, 0.5) showed similar behavior. First, we found that when the networks did synchronize, they synchronized to solutions of the self-coupled node with delay with an identical *W*^*E*^ value (Figures 1J,K), given by Equations 5–7. This is indeed, similar to the instantaneously coupled network case where networks with a coupling strength of *W*^*E*^ can synchronize to solutions of Equations 5–7 with ϵ = 0 (Nicola et al., 2018; Nicola and Campbell, 2021).

However, the delay-coupled network did exhibit differences from the instantaneously coupled network, in both the synchronization and the nature of the attractors. For example, the ring network considered in Figures 1F–H would desynchronize at different parameter values (e.g., smaller rings) in the delay-coupled case vs. the instantaneous case. As the delay was increased further, smaller networks could desynchronize. In contrast, the randomly-coupled networks remained synchronized for all parameter values and delay values we considered. Thus, the preliminary simulations display some link to qualitative behaviors of the instantaneous case (synchronization to the self-coupled node) but with differences in the behavior of the delayed vs. non-delayed networks for otherwise identical parameter values.

###  The Single, Delay Coupled Node

**Figure 2:**
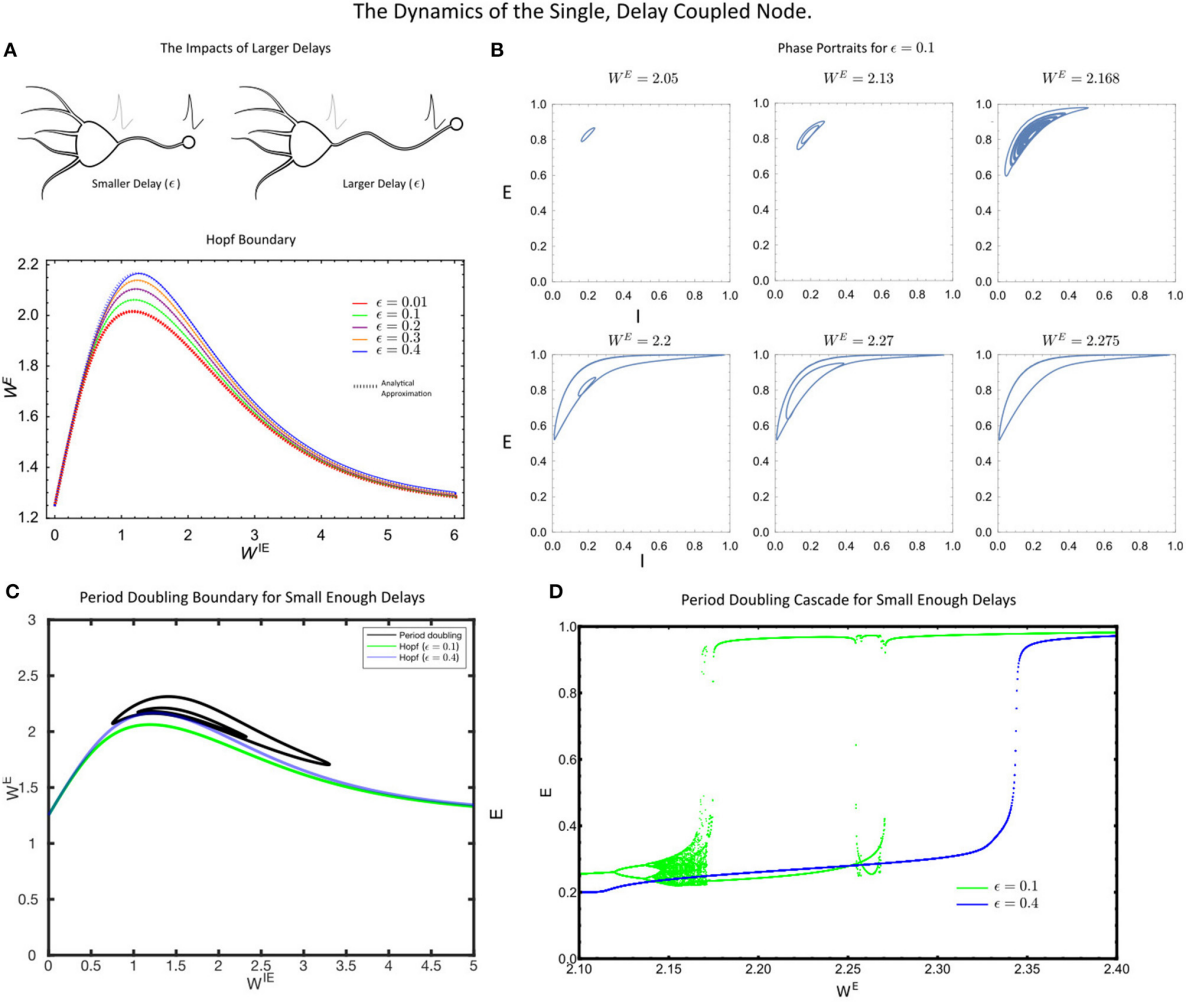
**(A)** The Hopf bifurcation boundary for the single, delayed self-coupled node, estimated analytically (dashed coloured lines) via a perturbation theory and numerically (solid lines, DDE-Biftool). **(B)** The phase portraits in the (*E*(*t*), *I*(*t*)) space for the single node for increasing values of *W*^*E*^. **(C)** The single self-coupled node undergoes period-doubling bifurcations for sufficiently small delay, (ϵ = 0.1). **(D)** A period-doubling cascade is present for small delays (ϵ = 0.1, green) but not large delays (ϵ = 0.4, blue). For all simulations, the parameter values were: *p* = 0.2, τ_1_ = 1, τ_2_ = 5, *W*^*IE*^ = 1 and *a* = 5.

Given the synchronization to the delayed, self-coupled node in Figure 1, we sought to investigate the bifurcation structure of the corresponding model (Equations 5–7). First, we found that as in the instantaneously coupled case, the self-coupled node displayed a supercritical Hopf bifurcation at a critical value of the coupling strength parameter *W*^*E*^ (Figure 2A). As *W*^*E*^ is increased, this Hopf bifurcation is followed by a period-doubling cascade to chaos (Figures 2B–D) provided that the delay is not too large. These results were confirmed using numerical simulation, numerical bifurcation analysis and by analytically approximating the Hopf-bifurcation curve (see Supplementary Materials Section 2).

As the delay, ϵ, in Equation 5–7 increased, we found that the critical value of the coupling strength *W*^*E*^ required to induce a Hopf bifurcation increased, thereby pushing the system into the more strongly coupled regime (Figure 2A). At the level of the single node, this is the primary factor that can eliminate the rich single node dynamics. In particular, for sufficiently large delays, the period doubling cascade is eliminated (Figure 2D), with the only remaining dynamics being a putative Canard-type explosion in limit cycle amplitude (see Nicola et al., 2018). Thus, for small delays, the single self-coupled node maintains many of the rich dynamical states of the instantaneously coupled system. However, for sufficiently large delay in the self-coupling, the rich-dynamical repertoire of the single node system is largely eliminated as the Hopf-bifurcation is only induced at strong coupling (*W*^*E*^) values.

###  Master Stability Function Analysis of the System With Delays

**Figure 3:**
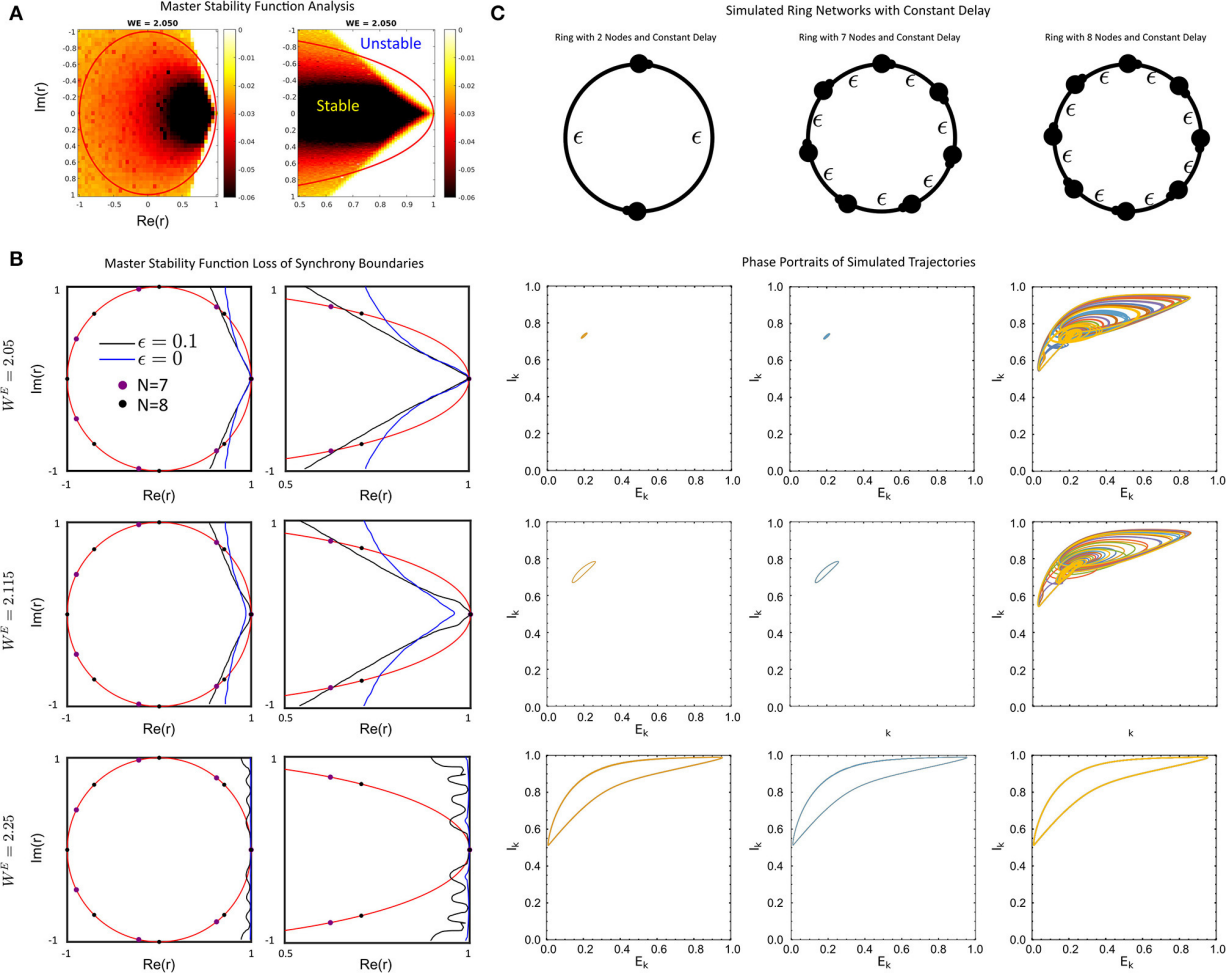
**(A)** The full Master-Stability Function (MSF) computed for *W*^*E*^ = 2.05 and ϵ = 0.1. **(B)** The sign-change boundaries for the MSF for no delay (blue) and delay ϵ = 0.1 (black) with *W*^*E*^ = 2.05 (top), *W*^*E*^ = 2.115 (middle), *W*^*E*^ = 2.25 (bottom) for the full unit-circle region (left) and a zoom (right). **(C)** Simulated ring networks for *N* = 2 (left), *N* = 7 (middle) and *N* = 8 (right) rings with the values of *W*^*E*^ as in **(B)**. For all simulations, the parameter values were: ϵ = 0.1, *p* = 0.2, τ_1_ = 1, τ_2_ = 5, *W*^*IE*^ = 1, and *a* = 5.

With the dynamics of the single self-coupled node largely resolved, we sought to determine how networks would synchronize to non-equilibrium (e.g., limit cycle or chaotic attractor) solutions. First, we applied the Master Stability Function approach (MSF) for the system with a constant fixed delay (Figure 3A, see Methods). Briefly, the Master Stability Function, λ(*r*), is a function which is evaluated at the eigenvalues of a connectivity matrix. If λ(*r*_*i*_) < 0 for all *i* = 1, 2, …*N* eigenvalues, then synchronized solutions are stable for any matrix with eigenvalues *r*_1_, *r*_2_, …*r*_*N*_. If, however, λ(*r*_*i*_) > 0 for any *i*, then the synchronized solution is unstable.

First, we find that for a fixed delay, the change in the MSF in the delay-coupled vs instantaneously coupled case is dependent on the connectivity matrix and global coupling strength *W*^*E*^. In particular, connectivity matrices with complex-eigenvalues that are large in magnitude with postive real parts are likely to lose stability of the synchronized solution when the network communication is delayed, as opposed to when it is instantaneous (Figure 3B top, middle). In contrast, connectivity matrices with purely real eigenvalues, as is the case with symmetric matrices, can gain stability (Figure 3B, middle). This is the differential impact of the delay on the connectivity.

**Figure 4:**
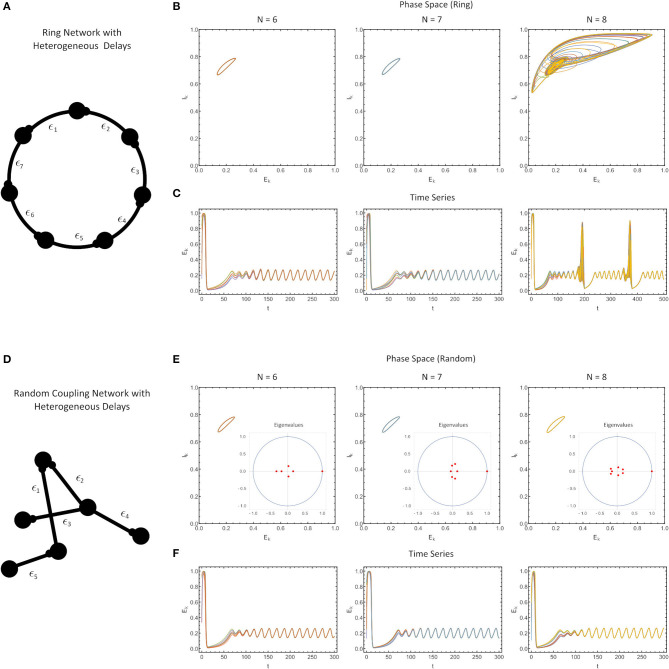
**(A)** Ring network with heterogeneous delays, where each delay is drawn from a beta-distribution (see Methods) with the finite sample also renormalized to have a sample mean of ϵ = 0.1. **(B)** Phase portrait of the ring in [*E*(*t*), *I*(*t*)] space, for *N* = 6 (left), *N* = 7 (middle) and *N* = 8 (right). **(C)** The time series of simulations. **(D)** A randomly-coupled network with heterogeneous delays, drawn as in **(A–C)**. **(E)** The phase portrait in the [*E*(*t*), *I*(*t*)] space with the eigenvalue spectrum of the sample-weight matrix drawn as an inset for randomly-coupled networks with *N* = 6 (left), *N* = 7 (middle) and *N* = 8 (right). **(F)** Time series of the simulations. For all simulations, the parameter values were: *W*^*E*^ = 2.115, *p* = 0.2, τ_1_ = 1, τ_2_ = 5, *W*^*IE*^ = 1, and *a* = 5.

An example of the former situation is a uni-directional ring. The spectrum of the connectivity matrix in this case lies on the unit circle and the second largest eigenvalue increases as the size of the ring increases. Thus delay will tend to destabilize larger networks before smaller networks. This can be seen in Figure 3B where the eigenvalues for unidirectional rings with *N* = 7 and *N* = 8 are displayed with the MSF. The MSF analysis predicts that both networks will be synchronized for ϵ = 0, but the larger network can be desynchronized for large enough delay. This was verified using numerical simulations of the full network (Figure 3C), where the ring of *N* = 8 nodes is desynchronized by the delay while that with *N* = 7 is not. Networks with random coupling also have complex eigenvalues, but the distribution tends to be clustered near the origin, especially for larger networks. See Figure 4E for some example distributions. Thus, for our model, these networks should exhibit synchronized solutions, largely unaffected by the presence of delays. Numerical simulations of some specific networks confirm this (see Supplementary Figure 1).

An example of a symmetric network is a lattice. Here the size of the second largest eigenvalue increases with the size of the network *N*. It was shown in Nicola and Campbell (2021) that for the model (Equation 1) with no delay (ϵ = 0) and *W*^*E*^ = 2.115 a lattice of *N* = 15 nodes is synchronized while that with *N* = 16 is desynchronized. Figure 3B indicates that with delay ϵ = 0.1 and the same value of *W*^*E*^ the lattice will be synchronized up to much larger values of *N*.

###  Heterogeneous Delays Largely Mirror Homogeneous Delay Case

Finally, we investigated how robust our results would be if the delays in our network were not homogeneous, but different for each connection. Here, the MSF approach does not extend, and thus, we opted to use numerical simulations for certain simple connectivity matrices (Figure 4). The only constraints in constructing these networks were that 1) all delays generated were positive and drawn from a beta-distribution and 2) the delays were re-scaled to force the sample mean of the delay to exactly match the nominal delay value we considered in Figures 1–3 (ϵ = 0.1).

First, we found that for ring networks, heterogeneity in the delays does not appreciably alter the synchronization characteristics of the network for the same fixed value of the coupling strength (*W*^*E*^) as in the homogeneous delay network (Figures 4A–C). In fact, even the attractors themselves were minimally altered (compare Figures 3C, 4B).

Second, we found that for all-to-all connected, row-sum normalized randomly-coupled networks, the solutions once again synchronized to identical attractors as for the ring networks (Figures 4C,F). Note that for systems which are all-to-all coupled, and with randomly chosen, row-sum normalized, the eigenvalues of the connectivity matrix shrink with the network size (aside from the dominant eigenvalue), which is a consequence of random matrix theory (Pastur and Shcherbina, 2011).

Of course the solutions cannot be perfectly synchronized since the delays are different. Close inspection shows that the different nodes have phase differences on the order of the size of the delay. Since the timescale of the delay is much smaller than the timescale of the oscillations in the WC system, these difference are not apparent in longer simulations. This can be explained by the analysis of Lücken et al. (2013) which determines conditions under which the distribution of delays in a network may be changed but still give equivalent dynamical behavior. The results of Lücken et al. (2013) apply directly to our ring networks and indicate that the system with heterogeneous delays will have the same attractor as that with homogeneous delays, but the phase relationships between the neurons will be different. A synchronized solution for the system with homogeneous delays becomes desynchronized in the system with heterogeneous delays, with the timescale of the desynchronization between neurons determined by the size of the delays.

Thus, numerically we find that the MSF results are robust for this WC system even with a heterogeneous distribution of delays, so long as the system with heterogeneous delays is compared to the homogeneous system with a delay equal to the sample mean of the heterogeneous system.

## 4. Discussion

The impact of delays on a network cannot be readily disentangled without simultaneously considering both the network topology, and the dynamics of individual nodes. Here, we considered all three in networks of delay-coupled, homoeostatically controlled Wilson-Cowan nodes with the Master Stability Function formalism. First, we find that when networks do synchronize, they synchronize to the single self-delay coupled node. The single node itself undergoes a Hopf-bifurcation to induce oscillations which requires a stronger global strength with larger delay. For small delays, the behavior of the network is similar to the non-delay coupled case, and to the behavior of other neural systems (see Keane et al., 2012 for example). For larger delays, the shift in the Hopf-bifurcation to stronger coupling values has a secondary impact: all mixed-mode, period doubled, and chaotic solutions are no longer present. Next, by applying the MSF approach, we found that the impacts of a delay are dependent on the network structure. Networks with large magnitude, complex eigenvalues (like rings) are likely to lose stability in their synchronous solution(s) while networks with large magnitude, purely real eigenvalues are likely to gain stability in their synchronous solutions. For a sufficiently large delay, which pushes up the global coupling strength necessary to induce oscillations, synchrony is the norm.

The size of delay in our study was chosen so that the ratio of the delay (ϵ) to the synaptic time constants was <1, as synaptic delays are typically in the sub-millisecond to millisecond range (Roxin et al., 2005; Ghosh et al., 2008; Deco et al., 2009). Nevertheless, delays in this biologically plausible range could still be large enough to induce the effects discussed above.

Our work highlights the importance of considering the network structure when considering the effect of time delay on synchronization behavior. In all cases we considered, the delay decreases the size of the region where synchronization is stable, however the region of stability also changes shape. In general, the region of stability near the right half of the unit circle decreases. This means that structured networks (such as unidirectional rings) are easier to desynchronize with larger delay. This is consistent with studies of structured networks that show that increasing the delay can lead to desynchronized cluster-like solutions (Choe et al., 2010; Kyrychko et al., 2014; Wang and Campbell, 2017; Kaslik and Mureşan, 2020; Kaslik et al., 2020). However, the synchronization region near the real axis was largely unchanged when the nodes exhibit periodic solutions. This means that networks with symmetric or near symmetric coupling are resistant to desynchronization by the delay. This is consistent with the results of studies across a variety of coupled networks with time delay (Dhamala et al., 2004; Choe et al., 2010; Flunkert et al., 2010, 2014; Kyrychko et al., 2014). For both the delayed and instantaneously coupled networks, the key determining factor for synchronization is the second-largest eigenvalue of the normalized connectivity matrix (Nicola and Campbell, 2021). Networks that generate larger eigenvalue distributions (e.g., more sparsely coupled networks) are more likely to desynchronize than networks that generate smaller eigenvalue distributions (e.g., more densely coupled networks).

A novel observation in our work was the influence of chaotic node behavior on synchronization. For networks with symmetric or near-symmetric coupling, a region of desychronization occurs when the nodes exhibit chaotic or irregular behavior. As discussed above, delays decrease the size of this region of desynchronization due to the fact that increasing the delay can destroy the chaotic behavior. If one considers coupling strengths were increasing the delay creates or preserves the chaotic behavior of the nodes then the delay can *increase* the size of the region of desynchronization. Nevertheless, we always observe the ultimate loss of the chaotic solutions for sufficiently large delay. This is a subtle effect of the model setup where the type of synchronized solution that occurs depends on the coupling strength.

The fact that time delays can influence synchronization behavior has long been understood (Crook et al., 1997; Ermentrout and Kopell, 1998; Ko and Ermentrout, 2007; Choe et al., 2010; Lehnert et al., 2011; Pérez et al., 2011; Dahms et al., 2012; Panchuk et al., 2013; Sun and Guofang, 2017). Here we have contributed to this understanding through our study of Wilson-Cowan networks with homeostatic adjustment of the inhibitory weight. Our work builds on and extends prior work on Wilson-Cowan networks with time delays, which focussed primarily on small networks (one or two nodes) and/or networks without the homeostatic adjustment (Coombes and Laing, 2009; Pasillas-Lépine, 2013; Kaslik and Mureşan, 2020; Kaslik et al., 2020).

## Data Availability Statement

The computer code for this study can be found on: ModelDB (https://senselab.med.yale.edu/modeldb/) Accession Number: 267010.

## Author Contributions

IA-D, LC, and WN performed the numerical simulations. IA-D and SC performed the analysis. IA-D, LC, WN, and SC wrote the manuscript and Supplementary Materials. All authors contributed to the article and approved the submitted version.

## Conflict of Interest

The authors declare that the research was conducted in the absence of any commercial or financial relationships that could be construed as a potential conflict of interest.
