# Supplementary material for: The Impact of Small Time Delays on the Onset of Oscillations and Synchrony in Brain Networks
Source: Front Syst Neurosci. 2021 Jul 5;15:688517. doi: 10.3389/fnsys.2021.688517 (PMC8287421; doi:10.3389/fnsys.2021.688517)
Supplement: Supplementary file 1 [file Data_Sheet_1.PDF]

Supplementary Material

1 THE SYNCHRONOUS SOLUTION

Using the condition of identical delays

$$\epsilon_{kj} = \epsilon, \quad \forall k, j$$

in the model equation (1) from the main text we obtain

$$\begin{aligned} \tau_1 \frac{dE_k}{dt} &= -E_k + \phi\left(\textstyle\sum_{j=1}^{N} \boldsymbol{W}_{kj}^{EE} E_j(t-\epsilon) - W_k^{EI} I_k\right) \\ \frac{dI_k}{dt} &= -I_k + \phi(W^{IE} E_k) \\ \tau_2 \frac{dW_k^{EI}}{dt} &= I_k(E_k - p) \end{aligned} \tag{S1}$$

where $k = 1, \ldots, N$.

We look for a synchronous solution of this model, that is, a solution where all the nodes have identical behaviour:

$$(E_k, I_k, W_k^{EI}) = (E_s(t), I_s(t), W_s^{EI}(t)), \ k = 1, \ldots, N. \tag{S2}$$

Substituting this into equation (S1) gives

$$\begin{aligned} \tau_1 \frac{dE_s}{dt} &= -E_s + \phi\left(\textstyle\sum_{j=1}^{N} \boldsymbol{W}_{kj}^{EE} E_s(t-\epsilon) - W_s^{EI} I_s\right) \\ \frac{dI_s}{dt} &= -I_s + \phi(W^{IE} E_s) \\ \tau_2 \frac{dW_s^{EI}}{dt} &= I_s(E_s - p) \end{aligned} \tag{S3}$$

Applying the row sum condition

$$\sum_{j=1}^{N} \boldsymbol{W}_{kj}^{EE} = W^E, \quad k = 1, 2, \ldots N$$

equation (S3) reduces to N copies of the following

$$\begin{aligned} \tau_1 \frac{dE_s}{dt} &= -E_s + \phi\left(W^E E_s(t-\epsilon) - W_s^{EI} I_s\right) \\ \frac{dI_s}{dt} &= -I_s + \phi(W^{IE} E_s) \\ \tau_2 \frac{dW_s^{EI}}{dt} &= I_s(E_s - p) \end{aligned} \tag{S4}$$

Dropping the subscript s, we see that this is the equation for a single node with delayed, recurrent coupling.

$$\begin{aligned}
\tau_1 \frac{dE}{dt} &= -E + \phi\left(W^E E(t-\epsilon) - W^{EI} I\right) \\
\frac{dI}{dt} &= -I + \phi\left(W^{IE} E\right) \\
\tau_2 \frac{dW^{EI}}{dt} &= I(E-p)
\end{aligned} \tag{S5}$$

Standard analysis shows that (S5) has the equilibrium

$$(\bar{E}, \bar{I}, \bar{W}^{EI}) = \left(p, \phi(W^{IE}p), \frac{W^E p - \phi^{-1}(p)}{\phi(W^{IE}p)}\right), \tag{S6}$$

which leads to the following equilibrium solution of the model (S1)

$$(\bar{E}_k, \bar{I}_k, \bar{W}_k^{EI}) = \left(p, \phi(W^{IE}p), \frac{W^E p - \phi^{-1}(p)}{\phi(W^{IE}p)}\right), \; k = 1, \dots N. \tag{S7}$$

It can be shown using an approach similar to Section 3 that the characteristic equation of the linearization of (S1) about the equilibrium (S7) is

$$\prod_{k=1}^{N} C_k(\lambda) = \prod_{k=1}^{N} \det(\mathbf{A}_k - \lambda \mathbf{I}) = 0$$

where $\mathbf{I}$ is the 3×3 identity matrix,

$$\mathbf{A}_k = \begin{pmatrix} -\tau_1^{-1} + \tau_1^{-1}\hat{r}_k \bar{M}_1 e^{-\lambda\epsilon} & -\tau_1^{-1}\bar{M}_1 \bar{W}^{EI} & -\tau_1^{-1}\bar{M}_1 \bar{I} \\ \bar{M}_2 & -1 & 0 \\ \tau_2^{-1}\bar{I} & 0 & 0 \end{pmatrix}$$

and $\bar{M}_1 = \phi'(\phi^{-1}(p)),\ \bar{M}_2 = W^{IE}\phi'(W^{IE}p)$. Thus

$$\begin{aligned}
C_k(\lambda) &= Q(\lambda) - \hat{r}_k \frac{\bar{M}_1}{\tau_1} \lambda(\lambda+1)e^{-\lambda\epsilon} \\
Q(\lambda) &= \lambda^3 + \lambda^2\left(\frac{1}{\tau_1} + 1\right) + \lambda\left(\frac{1}{\tau_1} + \frac{\bar{W}^{EI}\bar{M}_1\bar{M}_2}{\tau_1} + \frac{\bar{I}^2\bar{M}_1}{\tau_1\tau_2}\right) + \frac{\bar{I}^2\bar{M}_1}{\tau_1\tau_2}.
\end{aligned}$$

Since the connectivity matrix is assumed to have row sum W^E, it always has the eigenvalue W^E. We take $\hat{r}_1 = W^E$, which gives

$$C_1(\lambda) = Q(\lambda) - W^E \frac{\bar{M}_1}{\tau_1} \lambda(\lambda+1)e^{-\lambda\epsilon} = 0.$$

Straightforward calculations show that this corresponds to the characteristic equation of the linearization of the single node model (S5) about the equilibrium point (S6). It follows that any bifurcation of the

equilibrium (S6) in the single node system (S5) occurs for the synchronous equilibrium solution of the full model (S1).

2 PERTURBATION ANALYSIS OF THE HOPF BIFURCATION CURVE

We apply a perturbation method to the linearization of the single node model about the equilibrium (S6) to find the Hopf bifurcation curve equation in the (W^{IE}, W^{E}) parameter space.

As discussed above, the characteristic equation of the linearization of (S5) about the equilibrium (S6) is

$$\Delta(\lambda;\epsilon) := Q(\lambda) - P(\lambda;\epsilon) = 0 \tag{S8}$$

where

$$Q(\lambda) = \lambda^3 + q_2\lambda^2 + q_1\lambda + q_0 \qquad \text{and} \qquad P(\lambda;\epsilon) = p_0\lambda(\lambda+1)e^{-\lambda\epsilon} \tag{S9}$$

with

$$q_2 = \frac{1}{\tau_1} + 1, \quad q_1 = \frac{1}{\tau_1} + \frac{\bar{W}^{EI}\bar{M}_1\bar{M}_2}{\tau_1} + \frac{\bar{I}^2\bar{M}_1}{\tau_1\tau_2}, \quad q_0 = \frac{\bar{I}^2\bar{M}_1}{\tau_1\tau_2}, \quad p_0 = W^E\frac{\bar{M}_1}{\tau_1} \tag{S10}$$

where $\bar{M}_1 = \phi'(\phi^{-1}(p)),\ \bar{M}_2 = W^{IE}\phi'(W^{IE}p)$.

THEOREM 2.1. *When $\epsilon = 0$, the equilibrium is locally asymptotically stable if*

$$W^E\bar{M}_1 < 1. \tag{S11}$$

PROOF. At $\epsilon = 0$, the characteristic equation becomes

$$\Delta(\lambda;0) := \lambda^3 + (q_2 - p_0)\lambda^2 + (q_1 - p_0)\lambda + q_0 = 0 \tag{S12}$$

By the Routh-Hurwitz stability criterion, all roots of (S12) have negative real part if $q_2 - p_0 > 0$, $q_0 > 0$ and $(q_2 - p_0)(q_1 - p_0) - q_0 > 0$. It is clear that $q_0 > 0$.

When (S11) holds, we have

$$q_2 - p_0 = \frac{1}{\tau_1}\Big(1 + \tau_1 - W^E\bar{M}_1\Big) > 0$$

Furthermore, we have

$$\begin{aligned}(q_2 - p_0)(q_1 - p_0) - q_0 = &\frac{1}{\tau_1^2}\Big(1 + \tau_1 - W^E\bar{M}_1\Big)\Big(1 - W^E\bar{M}_1\Big) \\ &+ \frac{\bar{W}^{EI}\bar{M}_1\bar{M}_2}{\tau_1^2}\Big(1 + \tau_1 - W^E\bar{M}_1\Big) + \frac{\bar{I}^2\bar{M}_1}{\tau_1^2\tau_2}\Big(1 - W^E\bar{M}_1\Big) > 0.\end{aligned}$$

Thus, the equilibrium is locally asymptotically stable when (S11) holds.

Let $\epsilon > 0$ and suppose $\lambda = I\omega$ $(\omega > 0)$ is a purely imaginary root of (S8). Substituting $\lambda = I\omega$ into (S8) and separating the real and imaginary parts of $\Delta(I\omega;\epsilon) = 0$, we obtain:

$$\begin{aligned} q_0 - q_2\omega^2 &= p_0\omega\sin(\omega\epsilon) - p_0\omega^2\cos(\omega\epsilon) \\ q_1\omega - \omega^3 &= p_0\omega^2\sin(\omega\epsilon) + p_0\omega\cos(\omega\epsilon). \end{aligned} \tag{S13}$$

Now we use perturbation analysis to find the Hopf bifurcation curve in the (W^{IE}, W^E) parameter space when the time delay ϵ is small ($\epsilon \ll 1$). Since q_1 and p_0 depend on W^E, we write

$$q_1 = q_{10} + q_{11}W^E \quad \text{and} \quad p_0 = p_{00}W^E$$

where

$$q_{10} = \frac{1}{\tau_1} + \frac{\bar{I}^2\bar{M}_1}{\tau_1\tau_2} - \frac{\bar{M}_1^2\bar{M}_2}{\tau_1\phi(W^{IE}p)}, \quad q_{11} = \frac{p\bar{M}_1\bar{M}_2}{\tau_1\phi(W^{IE}p)}, \quad p_{00} = \frac{\bar{M}_1}{\tau_1}.$$

Let $\omega = \omega_0 + \epsilon\omega_1 + \epsilon^2\omega_2 + \ldots$ and $W^E = W_0^E + \epsilon W_1^E + \epsilon^2 W_2^E + \ldots$. Then, the first equation of (S13) gives

$$\begin{aligned} q_0 - q_2\big(\omega_0 + \epsilon\omega_1 + \epsilon^2\omega_2 + \ldots\big)^2 &= p_{00}\left(W_0^E + \epsilon W_1^E + \epsilon^2 W_2^E + \ldots\right)\left(\omega_0 + \epsilon\omega_1 + \epsilon^2\omega_2 + \ldots\right) \\ &\big[\sin\left(\epsilon\omega_0 + \epsilon^2\omega_1 + \epsilon^3\omega_2 + \ldots\right) - \left(\omega_0 + \epsilon\omega_1 + \epsilon^2\omega_2 + \ldots\right)\cos\left(\epsilon\omega_0 + \epsilon^2\omega_1 + \epsilon^3\omega_2 + \ldots\right)\big] \end{aligned}$$

Hence,

$$\begin{aligned} & q_0 - q_2\left(\omega_0^2 + 2\epsilon\omega_0\omega_1 + \epsilon^2(\omega_1^2 + 2\omega_0\omega_2) + \ldots\right) \\ &\quad = p_{00}\left(W_0^E + \epsilon W_1^E + \epsilon^2 W_2^E + \ldots\right)\left(\omega_0 + \epsilon\omega_1 + \epsilon^2\omega_2 + \ldots\right) \\ &\quad \left[\left(\epsilon\omega_0 + \epsilon^2\omega_1 + \epsilon^3\omega_2 + \ldots\right) - \left(\omega_0 + \epsilon\omega_1 + \epsilon^2\omega_2 + \ldots\right)\left(1 - \frac{\left(\epsilon\omega_0 + \epsilon^2\omega_1 + \epsilon^3\omega_2 + \ldots\right)^2}{2}\right)\right] \end{aligned}$$

Thus,

$$\mathcal{O}(1): \ q_0 - q_2\omega_0^2 = -p_{00}\omega_0^2 W_0^E \tag{S14a}$$

$$\mathcal{O}(\epsilon): \ -2q_2\omega_0\omega_1 = p_{00}\omega_0^2 W_0^E - 2p_{00}\omega_0\omega_1 W_0^E - p_{00}\omega_0^2 W_1^E \tag{S14b}$$

$$\begin{aligned} \mathcal{O}(\epsilon^2): \ -q_2\left(\omega_1^2 + 2\omega_0\omega_2\right) = \frac{1}{2}p_{00}\Big(&\omega_1^4 W_0^E - 2\omega_1^2 W_0^E - 4\omega_0(\omega_2 W_0^E + \omega_1[W_1^E - W_0^E]) \\ &+ 2\omega_0^2[W_1^E - W_2^E]\Big) \end{aligned} \tag{S14c}$$

From the second equation of (S13), we have

$$\begin{aligned} &\Big(q_{10} + [W_0^E + \epsilon W_1^E + \epsilon^2 W_2^E + \ldots]q_{11}\Big)(\omega_0 + \epsilon\omega_1 + \epsilon^2\omega_2 + \ldots) - \\ &(\omega_0 + \epsilon\omega_1 + \epsilon^2\omega_2 + \ldots)^3 = p_{00}(W_0^E + \epsilon W_1^E + \epsilon^2 W_2^E + \ldots)\Big[(\omega_0 + \epsilon\omega_1 + \epsilon^2\omega_2 + \ldots) \\ &(\epsilon\omega_0 + \epsilon^2\omega_1 + \epsilon^3\omega_2 + \ldots) + 1 - \frac{(\epsilon\omega_0 + \epsilon^2\omega_1 + \epsilon^3\omega_2 + \ldots)^2}{2}\Big] \end{aligned}$$

Hence,

$$\mathcal{O}(1):\ q_{10}\omega_0 - \omega_0^2 + q_{11}\omega_0 W_0^E = q_{00}\omega_0 W_0^E \tag{S15a}$$

$$\mathcal{O}(\epsilon):\ -q_{10}\omega_1 - 3\omega_0^2\omega_1 + q_{11}\Big(\omega_1 W_0^E + \omega_0 W_1^E\Big) = p_{00}\Big(\omega_0^3 W_0^E + \omega_1 W_0^E + \omega_0 W_1^E\Big) \tag{S15b}$$

$$\begin{aligned}\mathcal{O}(\epsilon^2):\ & q_{10}\omega_2 - 3\omega_0\omega_1^2 - 3\omega_2\omega_0^2 + q_{11}\Big(\omega_2 W_0^E + \omega_1 W_1^E + \omega_0 W_2^E\Big) \\ &= p_{00}\Big(3\omega_0^2\omega_1 W_0^E + \omega_2 W_0^E + \omega_1 W_1^E + \omega_0 W_2^E + \omega_0^3 W_1^E - \frac{\omega_0^3}{2} W_0^E\Big)\end{aligned} \tag{S15c}$$

Notice that, the equations (S14a) and (S15a) represent the case with no delay. From Nicola et al. (2018), we have

$$\omega_0 = \sqrt{q_1 - p_0}$$

and

$$W_0^E = \frac{1}{\phi'\Big[\phi^{-1}(p)\Big]}\Big[1 - \tau_1\mu_+\Big].$$

where

$$\mu_+ = \frac{A_1(1 - A_2) - q_0 - 1 + \sqrt{[A_1(A_2 - 1) + q_0 + 1]^2 - 4A_1A_2[1 - A_1]}}{2(1 - A_1)}$$

with

$$A_1 = \frac{p\phi'(pW^{IE})W^{IE}}{\phi(pW^{IE})}, \quad A_2 = \frac{1 - p^{-1}\phi^{-1}(p)\phi'[\phi^{-1}(p)]}{\tau_1}.$$

From (S14b), we have

$$\omega_1 = \frac{p_{00}\omega_0(W_0^E + W_1^E)}{2(q_2 - p_{00}W_0^E)}.$$

Substituting ω_1 in (S15b) and solving for W_1^E leads to

$$W_1^E = \frac{Bq_{10}W_0^E + p_{00}\omega_0^3 W_0^E + Bq_{11}\big(W_0^E\big)^2 - 3\omega_0^2 W_0^E - p_{00}\big(W_0^E\big)^2}{Bq_{10} - p_{00}\omega_0 + q_{11}\omega_0 + Bq_{11}W_0^E - 3\omega_0^2 - p_{00}W_0^E}$$

where

$$B = \frac{p_{00}\omega_0}{2(q_2 - p_{00}W_0^E)}.$$

From (S14c), we have

$$\omega_2 = \frac{2p_{00}(\omega_1^2 W_0^E - \omega_0^2 W_1^E + \omega_0^2 W_2^E) + 4p_{00}\omega_0\omega_1(W_1^E - W_0^E) - p_{00}\omega_0^4 W_0^E - 2q_2\omega_1^2}{4\omega_0(q_2 - p_{00}W_0^E)}.$$

Substituting ω_2 in (S15c) and solving for W_2^E, we have

$$\begin{aligned}W_2^E = \frac{1}{Bq_{10} - p_{00}\omega_0 + q_{11}\omega_0 + Bq_{11}W_0^E - 3\omega_0^2 - p_{00}W_0^E}\Bigg(& 3\omega_0\omega_1^2 - \frac{1}{2}p_{00}\omega_0^3 W_0^E \\ & + 3p_{00}\omega_0^2\omega_1 W_0^E + p_{00}\omega_0^3 W_1^E + p_{00}\omega_1 W_1^E - q_{11}\omega_1 W_1^E\end{aligned}$$

$$+ B\Big[q_{10}W_1^E + q_{11}W_0^E W_1^E + \frac{1}{2}q_{10}\omega_0^2 W_0^E - \frac{3}{2}\omega_0^4 W_0^E - 6\omega_0\omega_1 W_0^E$$
$$+ 3\omega_1^2 W_0^E - \frac{1}{2}p_{00}\omega_0^2\Big(W_0^E\Big)^2 + \frac{1}{2}\omega_0^2 q_{11}\Big(W_0^E\Big)^2 - 3\omega_0^2 W_1^E$$
$$+ 6\omega_0\omega_1 W_1^E - p_{00}W_0^E W_1^E\Big] + C_1\Big[q_{10} - 3\omega_0^2 - p_{00}W_0^E + q_{11}W_0^E\Big]$$
$$+ C_2\Big[2\omega_0 q_{10}W_1^E - q_{10}\omega_1 W_1^E - 2\omega_0 p_{00}\Big(W_0^E\Big)^2 + 2\omega_0\omega_1\Big(W_0^E\Big)^2$$
$$+ p_{00}\omega_1\Big(W_0^E\Big)^2 - q_{11}\omega_1\Big(W_0^E\Big)^2 - 2\omega_0 q_{10}W_1^E + 2\omega_0 p_{00}W_0^E W_1^E - 2\omega_0 q_{11}W_0^E W_1^E\Big]\Bigg).$$

where

$$C_1 = \frac{q_2\omega_1^2}{2\omega_0(q_2 - p_{00}W_0^E)}, \qquad C_2 = \frac{p_{00}\omega_1}{2\omega_0(q_2 - p_{00}W_0^E)}.$$

For small time delay ϵ, the Hopf bifurcation curve in the (W^{IE}, W^E) parameter space is given by

$$W^E = W^E_{\text{Hopf}}\Big(W^{IE}\Big) := W_0^E\Big(W^{IE}\Big) + \epsilon W_1^E\Big(W^{IE}\Big) + \epsilon^2 W_2^E\Big(W^{IE}\Big). \tag{S16}$$

3 MASTER STABILITY FUNCTION ANALYSIS

To derive the Master Stability Function, we consider the linearization of the full model (S1) about the synchronous solution (S2).

Let $x_k = E_k - E_s(t),\ y_k = I_k - I_s(t),\ z_k = W_k^{EI} - W_s^{EI}(t)$ and $\sum_{j=1}^N W_{kj}^{EE} = W^E$. Then the linearization about the synchronous solution will be

$$\begin{aligned} \tau_1\frac{dx_k}{dt} &= -x_k + M_{s1}(t)\left(\textstyle\sum_{j=1}^N W_{kj}^{EE}x_j(t-\epsilon) - {W_s}^{EI}(t)y_k - I_s(t)z_k\right) \\ \frac{dy_k}{dt} &= -y_k + M_{s2}(t)x_k \\ \tau_2\frac{dz_k}{dt} &= (E_s(t) - p)y_k + I_s(t)x_k \end{aligned} \tag{S17}$$

where $M_{s1}(t) = \phi'(W^E E_s(t-\epsilon) - W_s^{EI}(t)I_s(t))$, $M_{s2}(t) = W^{IE}\phi'(W^{IE}E_s(t))$.

Let $\mathbf{x} = (x_1, \ldots, x_N)^T$, $\mathbf{y} = (y_1, \ldots, y_N)^T$, $\mathbf{z} = (z_1, \ldots, z_N)^T$. The linearization may be written in vector form as

$$\begin{aligned} \tau_1\mathbf{x}'(t) &= -\mathbf{x}(t) - M_{s1}(t)\left(W_s^{EI}(t)\mathbf{y}(t) + I_s(t)\mathbf{z}(t)\right) + \mathbf{W}^{EE}M_{s1}(t)\mathbf{x}(t-\epsilon) \\ \mathbf{y}'(t) &= -\mathbf{y}(t) + M_{s2}(t)\,\mathbf{x}(t) \\ \tau_2\mathbf{z}'(t) &= (E_s(t) - p)\mathbf{y}(t) + I_s(t)\mathbf{x}(t) \end{aligned}$$

Assume that $\mathbf{W}^{EE}$ is diagonalizable and $\mathbf{P}$ is an invertible matrix such that $\mathbf{P}^{-1}\mathbf{W}^{EE}\mathbf{P} = \text{diag}(\hat{r}_1, \hat{r}_2, \ldots, \hat{r}_N)$ where $\hat{r}_k \in \mathbb{C}$. Define

$$\eta_{\mathbf{x}} = \mathbf{P}^{-1}\mathbf{x},\ \eta_{\mathbf{y}} = \mathbf{P}^{-1}\mathbf{y},\ \eta_{\mathbf{z}} = \mathbf{P}^{-1}\mathbf{z}.$$

Then the linearization becomes

$$\begin{aligned}
\tau_1 \eta_{\mathbf{x}}{}'(t) &= -\eta_{\mathbf{x}}(t) - M_{s1}(t)\left(W_s^{EI}(t)\eta_{\mathbf{y}}(t) + I_s(t)\eta_{\mathbf{z}}(t)\right) + \mathbf{P}^{-1}\mathbf{W}^{EE}\mathbf{P}M_{s1}(t)\eta_{\mathbf{x}}(t-\epsilon) \\
\eta_{\mathbf{y}}{}'(t) &= -\eta_{\mathbf{y}}(t) + M_{s2}(t)\,\eta_{\mathbf{x}}(t) \\
\tau_2 \eta_{\mathbf{z}}{}'(t) &= (E_s(t) - p)\eta_{\mathbf{y}}(t) + I_s(t)\eta_{\mathbf{x}}(t)
\end{aligned}$$

This breaks up into N independent $3D$ systems

$$\begin{aligned}
\tau_1 \frac{d\eta_x}{dt} &= -\eta_x + M_{s1}(t)\left(\hat{r}_k \eta_x(t-\epsilon) - I_s(t)\eta_z - W_s^{EI}(t)\eta_y\right) \\
\frac{d\eta_y}{dt} &= -\eta_y + M_{s2}(t)\eta_x \\
\tau_2 \frac{d\eta_z}{dt} &= (E_s(t) - p)\eta_y + I_s(t)\eta_x
\end{aligned} \tag{S18}$$

where $\hat{r}_k$ are the eigenvalues of the connectivity matrix $\mathbf{W}^{EE}$.

REFERENCES

Nicola, W., Hellyer, P. J., Campbell, S. A., and Clopath, C. (2018). Chaos in homeostatically regulated neural systems. *Chaos: An Interdisciplinary Journal of Nonlinear Science* 28, 083104

SUPPLEMENTARY FIGURES

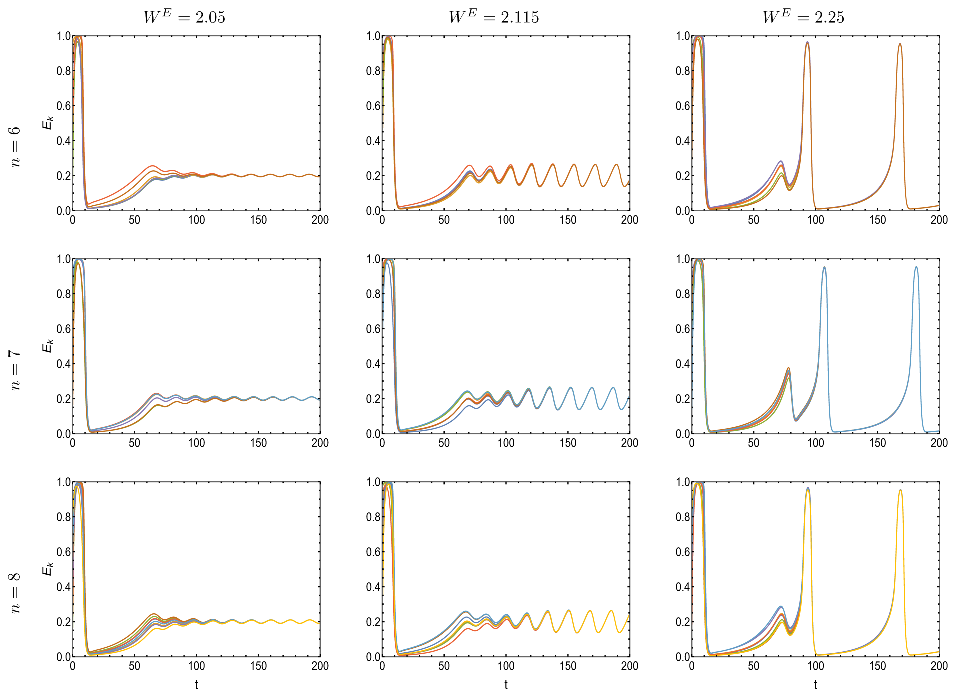

Figure S1. Numerical simulations of (S1) with random coupling and small delay ($\epsilon = 0.1$). Networks are all-to-all coupled with connection weights chosen randomly from a uniform distribution on $[0, 1]$. Eigenvalues of the connectivity matrices are shown in Figure 4 of the main text. Value of W^E and number of nodes is indicated. Other parameter values are: $p = 0.2,\ \tau_1 = 1,\ \tau_2 = 5, a = 5$ and $W^{IE} = 1$.
